# Assessment of the Relationship between Mortality and Troponin I Levels in Hospitalized Patients with the Novel Coronavirus (COVID-19)

**DOI:** 10.3390/medicina56120693

**Published:** 2020-12-13

**Authors:** Sinem Özyılmaz, Esra Ergün Alış, Emrah Ermiş, Samir Allahverdiyev, Hakan Uçar

**Affiliations:** 1 Department of Cardiology, Medical Faculty of Istanbul Aydin University, VM Florya Medical Park Hospital, Beşyol, Florya, Akasya Sk. No:4 D:1, 34295 Kucukcekmece, Istanbul, Turkey; emr_ermis@hotmail.com (E.E.); dr.samir.av@gmail.com (S.A.); ucarhakan2005@gmail.com (H.U.); 2 Department of Infection Disease 2, Medical Faculty of Istanbul Aydin University, VM Florya Medical Park Hospital, Beşyol, Florya, Akasya Sk. No:4 D:1, 34295 Kucukcekmece, Istanbul, Turkey; ergunesra03@yahoo.com

**Keywords:** novel, coronavirus disease 2019, cardiac damage, troponin I, mortality

## Abstract

*Background and Objectives*: This study aimed to evaluate the relationship between mortality and cardiac laboratory findings in patients who were hospitalized after a positive PCR for COVID-19 infection. *Materials and Methods*: This study included patients who were admitted to or referred to the hospital between 20 March and 20 June 2020, diagnosed with COVID-19 via a positive RT-PCR from nasal and pharyngeal swab samples. The troponin I level was measured from each patient. Medical records of patients were retrospectively reviewed and analyzed. *Results*: A hundred and five patients who were diagnosed with COVID-19 and hospitalized, or who died in the hospital due to COVID-19, were included in this study. There was a statistically significant difference between the troponin I high and low level groups in terms of age (years), BMI, shortness of breath (SB), oxygen saturation (%), hypertension, length of stay in the ICU; and for mortality, C-reactive protein, the neutrophil-to-lymphocyte ratio, hemoglobin, lactate dehydrogenase, ferritin, D-dimer, creatine kinase-MB, prothrombin time, calcium, and 25-hydroxy vitamin 25(OH)D3 (all *p* < 0.05). In the logistic analyses, a significant association was noted between troponin I and the adjusted risk of mortality. A ROC curve analysis identified troponin I values > 7.8 pg/mL as an effective cut-off point in mortality for patients with COVID-19. A troponin I value of higher than 7.8 pg/mL yielded a sensitivity of 78% and a specificity of 86%. *Conclusions*: The hospital mortality rate was higher among patients diagnosed with COVID-19 accompanied by troponin levels higher than 7.8 pg/mL. Therefore, in patients diagnosed with COVID-19, elevated troponin I levels >7.8 pg/mL can be considered an independent risk factor for mortality.

## 1. Introduction

Coronaviruses can infect various animals and humans, causing respiratory, enteric, cardiovascular, and neurological diseases. The COVID-19 infection caused by the coronavirus has spread from the Wuhan province of China since December 2019 and is responsible for the pandemic worldwide, causing morbidity and mortality [1]. Mortality increases with comorbidities such as cardiovascular disease, hypertension, diabetes, chronic lung disease, malignancy, male gender, and advancing age [2]. The novel type of coronavirus disease, COVID-19, with higher infectivity, can lead to severe pneumonia, pulmonary edema, acute respiratory distress syndrome (ARDS), or multiple organ failures and death [2,3]. Recent studies have highlighted that cardiac damage occurs with COVID-19-associated high troponin levels [1,2,3]. The rates of cardiac arrhythmia associated with high troponin levels in hospitalized patients, and the cardiac damage leading to cardiac death, have been reported to be between 7.2% and 59.6% [2,4,5,6,7,8,9].

In this study, done on hospitalized patients with a positive real-time reverse transcriptase–polymerase chain reaction (RT-PCR) diagnosis of COVID-19, we aimed to evaluate the association between troponin I levels and overall mortality.

## 2. Materials and Methods

### 2.1. Study Design and Patients

This study included 105 patients who were admitted or referred to the hospital between March 20 and 20 June 2020, and who were diagnosed with COVID-19 via a positive RT-PCR from nasal and pharyngeal swab samples, according to the guidelines of the Ministry of Health. This research was designed as a single-center, retrospective, observational study at Aydin University Medical Faculty Hospital, which is accepted as a COVID-19 pandemic hospital. The files of the patients were analyzed retrospectively, and all data were recorded. Informed consent forms were obtained from patients and their relatives. Approval to conduct the study was obtained from the Ministry of Health and the ethics committee of the hospital (ethics committee decision number 2020/282, approved date 2020.07.30). This work was compatible with the 1975 Helsinki Declaration.

### 2.2. Data Collection

The medical records of the patients’ files were reviewed to collect the data. Patients’ demographic data, medical histories, physical examination findings, laboratory results, treatment protocols (antiviral, antibiotic, and other), and length of hospitalization were recorded and analyzed.

### 2.3. Exclusion Criteria

We did not include patients with troponin I elevation due to an ischemic etiology. We excluded the patients with symptoms of myocardial ischemia, ischemic electrocardiographic (ECG) changes, or pathological Q waves on their ECGs. In patients that we were not sure about their diagnosis, we performed transthoracic echocardiography. The patients with a regional wall motion abnormality in a pattern consistent with an ischemic etiology were not included in the study. Patients with myocarditis due to unknown causes other than COVID 19, and individuals under the age of 18 or patients who did not accept treatment based on the current guidelines, or who did not want their data to be used, also were not included in the study.

### 2.4. Statistical Analyses

The study’s population was divided into two groups based on troponin I levels: the first group was high troponin I (>7.8 ng/mL, *n* = 21), and the second group was low troponin I (<7.8 ng/mL, *n* = 84). Using receiver operating characteristics (ROC) analysis, the troponin I cut-off value was determined to be 7.8 ng/mL for mortality. Continuous variables were expressed as the mean ± standard deviation; categorical variables were defined as percentages. The normally distributed continuous variables were assessed using the Kolmogorov–Smirnov test. A comparison of the parametric values of the two groups was performed using a two-tailed Student’s *t*-test, and for the nonparametric values, the Mann–Whitney U test. The categorical variables were compared using the odds ratio, chi-square test, or Fisher’s exact test. Pearson’s correlation coefficient was used to compare the parametric values of the two groups, and the Spearman correlation coefficient was used for the nonparametric comparisons. A backward stepwise logistic regression analysis, which included variables with *p* < 0.1, was performed to identify the independent predictors of mortality during hospitalization. The accuracy of the relevant variables from the regression analysis, to differentiate between the high troponin I and low troponin I groups, was assessed with the ROC curves to determine the area under the curve and optimal sensitivity and specificity. The survival curve for COVID-19 patients, grouped according to troponin levels, was constructed using the Kaplan–Meier method, with differences assessed by log-rank tests. Statistical evaluation of the data was performed using SPSS for Windows Version 22.0 software (SPSS Inc., Chicago, IL, USA). A *p*-value of <0.05 was considered statistically significant.

## 3. Results

### 3.1. Baseline Clinical Features on Admission

To predict clinical outcome, the initial test results were used rather than the highest value during hospitalization. Among the total 105 COVID-19 patients included in the study, the median age was 45 (20–87) years; 76 were male, 29 were female, 96 were alive, and 9 were dead. The baseline clinical features of the two groups (high and low troponin I groups) are summarized in Table 1. There was a statistically significant difference between the two groups in terms of age (years), BMI (kg/m^2^), heart rate (/min), systolic and diastolic blood pressure (mmHg), shortness of breath, O_2_ saturation (%), hypertension, stay in the intensive care unit, and mortality (all *p* < 0.05). The mean length of hospital stay was 11.12 ± 7.17 days in all patients, 12.90 ± 10.81 in the high troponin I group, and 10.67 ± 5.94 days in the low troponin I group. The number of patients followed up with in the intensive care unit was 15; the duration of follow-up in intensive care was 10.1 ± 3.21 in the high troponin I group (*n* = 10), 15.2 ± 11.3 days in the low group (*n* = 5), and 11.8 ± 10.4 days in total. Most of the patients had fever (>38 °C; *n* = 87), weakness (*n* = 87), and joint pain (*n* = 81). In addition, 22% of patients had hypertension (*n* = 11 for the high troponin I group; *n* = 12 for the low troponin I group). Chest computed tomography images showed that all patients had typical signs of COVID-19 involvement.

### 3.2. Medical Therapies

Hydroxychloroquine treatment was initiated in all patients who were diagnosed with COVID-19 by RT-PCR and hospitalized, and azithromycin treatment was initiated in 103 patients. As antiviral treatment, five of the patients were given lopinavir/ritonavir, 97 were given oseltamivir, and 15 were given favipiravir. Ceftriaxone was started in 73 patients thought to need antibiotics. As vitamin supplements, 99 were given D vitamins, and 97 were given C vitamins. Low molecular weight heparin treatment was applied to 90 patients for anticoagulant therapy. A plasma exchange procedure was applied to two patients, and extracorporeal membrane oxygenation (ECMO) was applied to one patient. One patient, a nurse, was taken to ECMO and died at the age of 30 after being followed for 20 days in ICU (10 days in ECMO). COVID-19 had been detected during her pregnancy, and the patient developed ARDS after the birth. Plasma exchange was also applied to this patient. The other patient who had plasma exchange was discharged as healthy. There was a statistically significant difference between the high and low troponin I groups in terms of the favipiravir and plasma exchange treatments (*p* <0.05).

### 3.3. Laboratory Findings on Admission

The laboratory findings of the two groups (high and low troponin I) are summarized in Table 1. There was a statistically significant difference between the groups in terms of blood urea nitrogen, creatinine, glomerular filtration rate (GFR), C-reactive protein (CRP), white blood cell count, neutrophil count, lymphocyte count, neutrophil-to-lymphocyte ratio (NLR), hemoglobin, lactate dehydrogenase, ferritin, D-dimer, creatine kinase-MB (CK-MB), prothrombin time (PT), calcium, and 25-hydroxy vitamin 25(OH)D3 (all *p* < 0.05).

### 3.4. Results Regarding the Relationship between Troponin and Mortality

**Figure 1:**
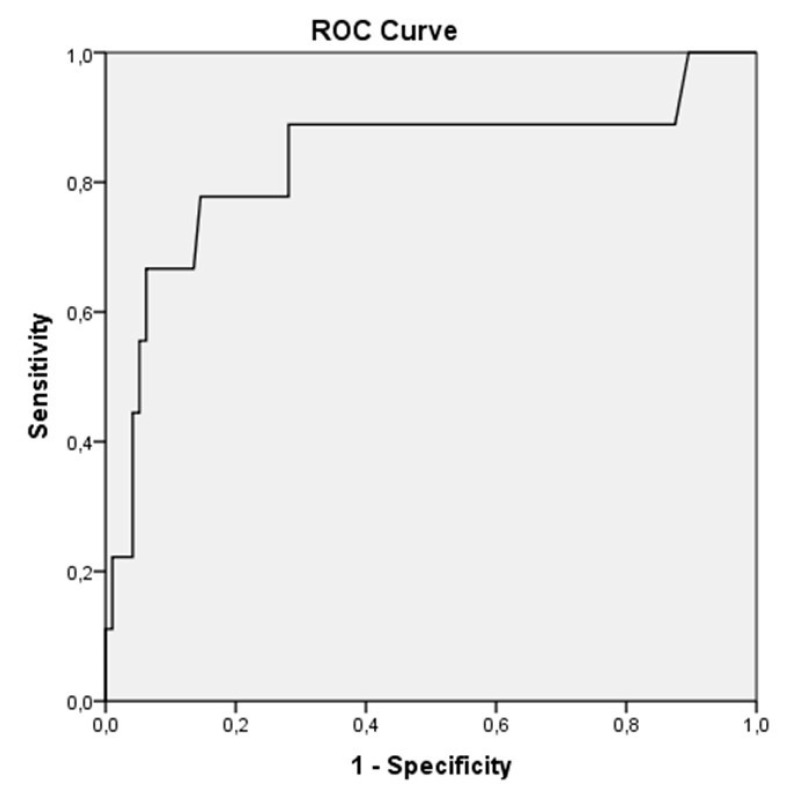
In the receiver operating characteristic (ROC) curve analysis, a troponin I > 7.8 pg/mL was identified as an effective cut-off point in the COVID 19 patients (area under curve = 0.832. 95% CI = 0.654–1.009. *p* = 0.001). A troponin I value of more than 7.8 pg/mL yielded a sensitivity of 78% and a specificity of 86%.

In this study, the mortality ratio was determined to be 8.5% (*n* = 9) during hospitalization. We performed univariate and multivariate analyses to determine the independent factors associated with mortality (Table 2 and Table 3). Troponin I, CRP, lymphocyte count, shortness of breath, hypertension, hyperlipidemia, diabetes mellitus, and coronary artery disease were analyzed using the multivariate regression model. Multivariate analyses with three different models were done by using backward selection for predicting mortality. All the parameters that were found to be significant in the univariate analysis were included into Model 1. Troponin I was found to be the most significant predictor of mortality. Model 2 consisted of parameters such as troponin I, CRP, shortness of breath, and lymphocyte count. Troponin I (*p* = 0.043) was found to be an independent significant predictor of mortality. Model 3, which consisted of the troponin I group and CRP, showed that troponin I (*p* = 0.009) was an independent significant predictor of mortality in patients with COVID-19. In the ROC curve analysis, a troponin I value >7.8 pg/mL was identified as an effective cut-off point in mortality for patients with COVID 19 (area under curve = 0.832, 95% CI = 0.654–1.009, *p* = 0.001). A troponin I value of >7.8 pg/mL yielded a sensitivity, specificity, positive, and negative predictive values of, 78%, 86%, 77%, and 85%, respectively (Figure 1).

**Figure 2:**
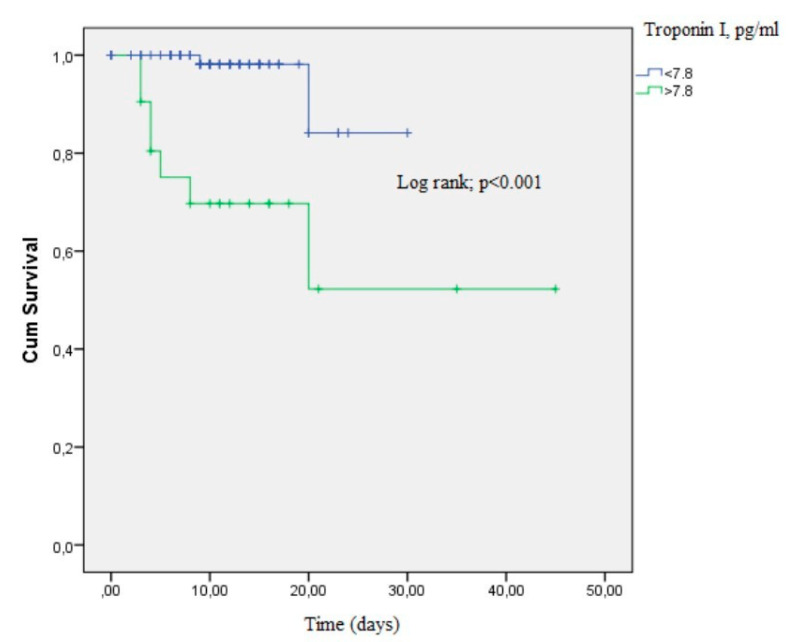
The Kaplan–Meier curve demonstrating survival of COVID-19 patients according to the troponin I groups (*p* < 0.001 by log-rank test).

The Kaplan–Meier curve for mortality according to the troponin I group (troponin I > 7.8 pg/mL, troponin I ≤ 7.8 pg/mL) in the entire population of patients is shown in Figure 2 (*p* < 0.001 by log-rank test).

## 4. Discussion

The most important novel result of this study is that a troponin I value above 7.8 pg/mL has a good sensitivity and specificity for predicting mortality in patients diagnosed with COVID-19.

In this study, in-line with previous studies, troponin levels were found to be higher in older patients and males [1,4]. Similar to the results of this study during the COVID-19 pandemic, the CK-MB and troponin levels were found to be high in patients who died due to COVID-19 and in patients who remained in the ICU [1,3,4,10,11,12,13,14]. In a study by Shi et al., the mortality rate was 51.2% in patients with high troponin levels and cardiac damage and 4.5% in patients without cardiac injury; Guo et al. reported these rates as 59.6% and 8.9%, respectively [1,4]. In this study, the mortality rate was 33% in hospitalized patients with high troponin levels and 2.4% in the low troponin group. The mortality rate in this study was lower than the mortality rate of the previous studies.

The elevation of troponin in COVID-19 patients can be explained by several possible mechanisms. These are (1) viral myocarditis, (2) cytokine-induced myocardial damage, (3) microangiopathy, and (4) unmasked coronary artery disease. The COVID-19 spike protein binds to angiotensin converting enzyme 2 (ACE2) receptors for entry into the cell [15]. ACE2 is highly present in the pericytes of adult human hearts [10]. In addition to entering the cell with ACE2, COVID-19 reduces the return of angiotensin II (Ang-II) to angiotensin 1–7 (Ang-1-7) by suppressing ACE2 expression. Ang 1–7 creates protective cardiovascular effects in target organs. No histopathological data on COVID-19 myocarditis have been published so far [10,16]. As a result, the suppression of ACE2 expression and subsequent increase in Ang-II levels may pose a threat to the heart and vessels in COVID-19 patients [10].

Endothelial dysfunction, cytokine storms, oxidative stress, and Ang-II upregulation may explain the common occurrence of coagulopathy in severe coronavirus disease [10]. Approximately half of patients with COVID-19 have high D-dimer levels, which is associated with disease severity and high mortality rates [17]. Guo et al. [4] showed that D-dimer levels were significantly higher in the group with high troponin levels than in the group with normal troponin levels. Similarly, in this study, D-dimer levels were found to be higher in the group with high troponin levels [18,19,20]. Huang et al. emphasized that the imbalance of T helper 1 and T helper 2 responses in patients with COVID-19 results in a cytokine storm and contributes to myocardial injury [5]. Cytokines, caused by circulating systemic inflammation, create type 1 MI, leading to thrombus formation, atherosclerotic plaque instability, and rupture [18]. There is very little data on MI-related symptoms and electrocardiogram changes in COVID-19 [1]. Interestingly, Guo et al. [4] reported that no patients showed acute MI findings at admission [4]. In this study, no MI findings were observed in any patient.

The reduction of Ang 1–7 as a result of ACE2 receptor downregulation caused by COVID-19 can increase cytokine storms, which may cause a severe inflammatory response related to myocardial damage and infection [4,10]. In the studies by Shi and Guo and Lin et al., a significant positive linear correlation was found between plasma troponin and CRP levels, suggesting that myocardial damage may be closely related to inflammatory pathogenesis [1,4,21]. Similarly, in this study, CRP values were found to be high in the high troponin I group. Guo and Lin et al. also showed that, in severe COVID-19 patients, the ferritin, LDH, and D-dimer levels were higher and lymphocyte counts were lower than in those without severe disease [4,21]. In addition to these results, Lin et al. [21] reported that ferritin was an independent risk factor for severe COVID-19 disease. Tatum [22] and Xia et al. [23] observed marked increases in NLR and lymphopenia in patients with severe COVID-19 infection or death due to COVID-19. In both studies, NLR was found to be an independent risk factor for mortality or severe COID-19 infection. In this study, the high ferritin and NLR values in the high troponin group are consistent with the results from these other studies. Similar to these studies, ferritin and NLR values were also found to be significantly higher in the group with higher troponin levels.

A statistical significance was found between troponin I values and percentage of shortness of breath (SOB). While 76% of patients with high troponin I had SOB, SOB was absent in 76% of patients with low troponin levels. According to a previous study, Zheng at al. reported that patients with shortness of breath/dyspnea were more likely to develop into critical illness or even die [24].

In the study of Shi et al., cardiac biomarkers were measured on admission were collected, including troponin I, CK-MB, and myohemoglobin. Patients were categorized according to the presence or absence of cardiac injury. Patients with cardiac injury had a higher rate of mortality, both from symptom onset and from admission [1]. Therefore, this study, similar to our study, shows that the troponin I value, which was measured on admission, can provide information about mortality in the early period.

Guo et al. observed that a number of systemic complications, such as acute respiratory distress syndrome, malignant arrhythmias, including ventricular tachycardia/ventricular fibrillation, acute coagulopathy, and acute kidney injury, were higher in patients with high troponin values measured while they were hospitalized [4]. Troponin elevation might be due to these complications. If we had used the values in the later days of hospitalization, we might have misjudged the cardiac involvement due to COVID 19. Therefore, we thought that the first troponin I value received on admission to the hospital would be more objective.

Troponin I elevations in COVID-19 infection are likely to reflect non-coronary disease and the cardio-inflammatory process rather than acute coronary disease, such as myocardial infarction [25]. Based on this idea, we did not include the patients with symptoms of myocardial ischemia, ischemic ECG changes, or pathological Q waves on their ECGs. In patients whose diagnosis we were not sure about, we performed echocardiography; the patients with regional wall motion abnormality in a pattern consistent with an ischemic etiology also were not included in the study.

### Study Limitations

The relatively low number of patients in the high and low troponin groups is this study’s primary limitation. The fact that it was a retrospective, single-center study, and that electrocardiography and echocardiography could not be performed for all patients due to the isolation restrictions regarding these patients, are other limitations. Further limitations include the fibrinogen, N-terminal pro-brain natriuretic peptide, procalcitonin, and erythrocyte sedimentation rate values being absent, as well as the relatively low number of patients who died, and no cancer patients being included.

## 5. Conclusions

Hospital mortality is higher in the novel COVID-19 patients, whose troponin levels are higher than 7.8 pg/mL compared to patients with troponin I levels lower than 7.8 pg/mL. In this study, the troponin value constituted an independent risk indicator for mortality when it was over the cut-off value of >7.8 pg/mL.

## Figures and Tables

**Table 1:** Baseline and clinical characteristics of the troponin I high and low groups.

Characteristics of Patients	All Patients (n-Mean ± SD or Median (min–max))		Troponin I High Group (>7.8 pg/mL) (n-Mean ± SD or Median (min–max))	Troponin I Low Group(<7.8 pg/mL) (n-Mean ± SD or Median (min–max))	*p*-Values
Number of patients (*n*)	105		21 (20%)	84 (80%)	
Male (*n*)	76		15 (71.4%)	61 (72.6%)	0.913
Female (*n*)	29		6 (28.6%)	23 (27.4%)	
Age (years)	45 (20–87)		60 (26–87)	45 (20–83)	0.005
Body mass index (kg/m^2^)	25 (21–38)		27 (21–32)	25 (21–38)	0.008
Heart rate (/min)	81 (60–128)		105 (71–128)	72 (60–108)	<0.001
Systolic blood pressure (mmHg)	130 (91–168)		138 (105–168)	127 (91–150)	<0.001
Diastolic blood pressure (mmHg)	75 (57–98)		80 (57–98)	71 (57–94)	0.017
Shortness of breath	(+)	36	16 (76.2%)	20 (23.8%)	<0.001
	(−)	69	5 (23.8%)	64 (76.2)	
Saturation (%)	94 (45–98)		85 (45–96)	94 (60–98)	<0.001
Diabetes mellitus	(+)	9	4 (19%)	5 (6%)	0.061
	(−)	96	17 (81%)	79 (94%)	
Hypertension	(+)	23	11 (52.4%)	12 (14.3%)	<0.001
	(−)	82	10 (47.6%)	72 (85.7%)	
Hyperlipidemia	(+)	6	3 (14.3%)	3 (3.6%)	0.058
	(−)	99	18 (85.7%)	81 (96.4%)	
Cigarette	(+)	24	8 (38.1%)	16 (19%)	0.063
	(−)	81	13 (61.9%)	68 (81%)	
Coronary artery disease	(+)	14	5 (21.8%)	9 (10.7%)	0.114
	(−)	91	16 (76.2%)	75 (89.3%)	
COPD	(+)	18	5 (23.8%)	13 (15.5%)	0.365
	(−	87	16 (76.2%)	71 (84.5%)	
Fever	(+)	87	20 (95.2%)	67 (79.8%)	0.092
	(−)	18	1 (4.8%)	17 (20.2%)	
Weakness	(+)	87	17 (81%)	70 (83.3%)	0.796
	(−)	18	4 (19%)	14 (16.7%)	
Joint pain	(+)	81	14 (66.7%)	67 (79.8%)	0.201
	(−)	24	7 (33.3%)	17 (20.2%)	
Hospitalization	(+)	95	21 (100%)	74 (88.1%)	0.206
	(−)	10	0	10 (11.9%)	
Stay at the intensive care unit	(+)	15	10 (47.6%)	5 (6%)	<0.001
	(−)	90	11 (52.4%)	79 (94%)	
Mortality	(+)	9	7 (33.3%)	2 (2.4%)	<0.001
	(−)	96	14 (66.7%)	82 (97.6%)	
Duration of hospitalization	11.12 ± 7.17		12.90 ± 10.81	10.67 ± 5.94	0.205
Duration of intensive care unit	15 (11.8 ± 10.4)		10 (10.1 ± 3.21)	5 (15.2 ± 11.3)	0.394
**Treatment (*n*)**					
Medical therapy					
▪ Lopinavir/Ritonavir	(+)	5	1 (4.8%)	4 (4.8%)	1.000
	(−)	100	20 (95.2%)	80 (95.2%)	
▪ Oseltamivir	(+)	97	19 (90.5%)	78 (92.9%)	0.713
	(−)	8	2 (9.5%)	6 (7.1%)	
▪ Favipiravir	(+)	15	9 (42.9%)	6 (7.1%)	<0.001
	(−)	90	12 (57.1%)	78 (92.9%)	
▪ Seftriaxone	(+)	73	15 (71.4%)	58 (69%)	0.832
	(−)	32	6 (28.6%)	26 (31%)	
▪ Hydroxychloroquine	(+)	105	21 (100%)	84 (100%)	-
	(−)	0	0	0	
▪ Azytromycine	(+)	103	21 (100%)	82 (97.6%)	0.475
	(−)	2	0	2 (2.4%)	
▪ Vitamin C	(+)	97	20 (95.2%)	77 (91.7%)	0.581
	(−)	8	1 (4.8%)	7 (8.3%)	
▪ Vitamin D	(+)	99	21 (100%)	78 (92.9%)	0.207
	(−)	6	0	6 (7.1%)	
▪ Low molecular weight heparin	(+)	90	18 (85.7%)	72 (85.7%)	1.000
	(−)	15	3 (14.3%)	12 (14.3%)	
▪ Other therapy					
▪ Plasma exchange	(+)	2	2 (9.5%)	0	0.038
	(−)	103	19 (90.5%)	84 (100%)	
▪ ECMO	(+)	1	1 (4.8%)	0	0.200
	(−)	104	20 (95.2%)	84 (100%)	
LDL cholesterol (mg/dl)	84.09 ± 49.77		69.42 ± 46.30	88.86 ± 50.44	0.207
HDL cholesterol (mg/dl)	38.93 ± 15.91		41.70 ± 20.77	38.14 ± 14.50	0.519
Triglyceride (mg/dl)	107.13 ± 51.95		104.75 ± 47.60	107.82 ± 52.56	0.856
Total cholesterol (mg/dl)	141.73 ± 63.31		135.00 ± 62.08	143.70 ± 64.29	0.679
Blood urea nitrogen	30.09 ± 21.66		47.28 ± 39.93	25.74 ± 10.28	<0.001
Creatinine	0.86 (0–1)		0.98 (0.63–3.74)	0.82 (0.49–2)	0.005
Glomerular filtration rate	98 (15–457)		88 (15–124)	13.2 (37–457)	0.004
ESR	28.27 ± 19.84		34.26 ± 18.46	26.70 ± 20.03	0.176
CRP	47.5 (0.01–319)		148 (5.1–273)	13.2 (0.01–319)	<0.001
White blood cell count	7.21 ± 4.12		9.05 ± 5.66	6.75 ± 3.53	0.021
Neutrophil count	4.1 (1.4–83)		8 (2.0–83)	3.56 (1.4–19.7)	<0.001
Lymphocyte count	1.1 (0.6–6.9)		0.81 (0.1–6.9)	1.28 (0.36–3.33)	0.007
NLR	3.09 (0.89–31)		11.1 (2.21–30)	2.5 (0.89–31)	<0.001
Hemoglobin	13 (8.5–41.7)		12 (9–15.5)	13.2 (8.5–41.7)	0.007
Platelet count	217.13 ± 169.78		181.771 ± 885.45	225.97 ± 183.88	0.288
Lactate dehydrogenase	329.80 ± 214.81		498.71 ± 362.77	287.07 ± 129.25	<0.001
Ferritin	354 (3.61–3258)		727 (48–2684)	291 (3.6–3258)	0.008
D Dimer	583 (0–8902)		2271 (271–8802)	422 (0–8902)	<0.001
Troponin (pg/mL)	2.6 (0–1774.5)		15.4 (8.6–1774.5)	1.9 (0–7)	<0.001
CK-MB	15.03 ± 20.00		23.55 ± 39.99	12.58 ± 6.97	0.046
CK	130.80 ± 183.19		107.62 ± 88.44	135.81 ± 198.37	0.698
AST	37.66 ± 29.36		30.52 ± 15.16	39.45 ± 31.76	0.214
ALT	41.64 ± 30.16		34.19 ± 20.49	43.51 ± 31.85	0.207
INR	1.53 ± 2.48		1.47 ± 0.92	1.56 ± 2.86	0.891
PT	15.3 ± 6.0		18.1 ± 10.6	14.3 ± 2.0	0.020
PTT	89.5 (8.7–110.8)		71.9 (8.7–109)	90.8 (34.3–110.8)	0.089
Sodium	138.07 ± 4.17		136.95 ± 5.66	138.35 ± 3.69	0.169
Potassium	3.98 ± 0.33		4.06 ± 0.45	3.95 ± 0.29	0.209
Calcium	8.5 (7.3–10)		7.95 (7.3–9.1)	8.6 (7.7–10)	<0.001
25-hydroxy vitamin 25 (OH) D3	16.5 (4.1–90)		8.15 (4.1–47)	18 (6–90)	<0.001

ALT: alanine aminotransferase, AST: aspartate aminotransferase, CRP: C- reactive protein, CK: Creatine kinase, CK MB: Creatine Kinase MB, COPD: chronic obstructive pulmonary disease, ECMO: Extracorporeal membrane oxygenation, ESR: Erythrocyte sedimentation rate, BMI: body mass index, DM: diabetes mellitus, HL: hyperlipidemia, HT: hypertension, INR: International Normalized Ratio, NLR: Neutrophil/Lymphocyte ratio, PT: Prothrombin time, PTT: partial thromboplastin time.

**Table 2:** Univariate analyses for independent high-risk predictors of mortality.

Variables	Odds Ratio	Confidence Interval (95%)	*p*-Values
Troponin I	20.500	3.857–108.960	< 0.001
C-Reactive Protein	1.012	1.003–1.021	0.008
Lymphocyte count	0.087	0.012–0.617	0.015
Shortness of breath	8.086	1.583–41.301	0.012
Hypertension	5.066	1.240–20.695	0.024
Hyperlipidemia	6.571	1.020–42.350	0.048
Diabetes mellitus	4.833	1.029–22.693	0.046
Coronary artery disease	6.880	1.584–29.888	0.010
Age	1.031	0.988–1.076	0.156
Gender	0.743	0.173–3.190	0.689
D dimer	1.000	1.000–1.000	0.080
Creatinine	1.137	0.219–5.908	0.879
Hemoglobin	0.799	0.543–1.176	0.256

**Table 3:** Multivariate analyses for independent high-risk predictors of mortality.

	Variables	Odds Ratio	Confidence Interval (95%)	*p*-Values
Model 1	Troponin I	10.364	0.792–135.680	0.075
	C-Reactive Protein	1.014	0.999–1.028	0.069
	Lymphocyte count	0.107	0.008–1.380	0.087
	Shortness of breath	2.891	0.176–47.556	0.457
	Hypertension	0.790	0.090–6.935	0.832
	Hyperlipidemia	4.060	0.011–1555.792	0.644
	Diabetes mellitus	2.806	0.067–117.047	0.588
	Coronary artery disease	0.024	0.000–1.207	0.062
Model 2	Troponin I	7.894	1.062–58.666	0.043
	C-Reactive Protein	1.009	0.998–1.021	0.123
	Shortness of breath	0.859	0.102–7.261	0.889
	Lymphocyte count	0.320	0.053–1.926	0.213
Model 3	Troponin I	10.302	1.772–59.902	0.009
	C-Reactive Protein	1.010	0.999–1021	0.079
